# HTLV-1 bZIP factor suppresses TDP1 expression through inhibition of NRF-1 in adult T-cell leukemia

**DOI:** 10.1038/s41598-017-12924-0

**Published:** 2017-10-09

**Authors:** Yoko Takiuchi, Masayuki Kobayashi, Kohei Tada, Fumie Iwai, Maki Sakurada, Shigeki Hirabayashi, Kayoko Nagata, Kotaro Shirakawa, Keisuke Shindo, Jun-ichirou Yasunaga, Yasuhiro Murakawa, Vinodh Rajapakse, Yves Pommier, Masao Matsuoka, Akifumi Takaori-Kondo

**Affiliations:** 1 Department of Hematology and Oncology, Graduate School of Medicine, Kyoto University, 54 Shogoin-kawaracho, Sakyo-ku, Kyoto 606-8507 Japan; 2 Laboratory of Virus Control, Institute for Frontier Life and Medical Sciences, Kyoto University, 53 Shogoin-kawaracho, Sakyo-ku, Kyoto 606-8507 Japan; 3 RIKEN Preventive Medicine and Diagnosis Innovation Program, 1-7-22 Suehiro-cho, Tsurumi-ku, Yokohama, Kanagawa 230-0045 Japan; 4 Developmental Therapeutics Branch and Laboratory of Molecular Pharmacology, Center for Cancer Research, National Cancer Institute, National Institutes of Health, Building 37, Room 5068, Bethesda, MD 20892-4255 USA; 5 Department of Hematology, Rheumatology and Infectious Disease, Kumamoto University Graduate School of Medicine, 1-1-1 Honjo, Chuo-ku, Kumamoto 860-8556 Japan

## Abstract

Adult T-cell leukemia (ATL) is an aggressive T-cell malignancy caused by human T-cell leukemia virus type 1 (HTLV-1). We recently reported that abacavir, an anti-HIV-1 drug, potently and selectively kills ATL cells. This effect was attributed to the reduced expression of tyrosyl-DNA-phosphodiesterase 1 (TDP1), a DNA repair enzyme, in ATL cells. However, the molecular mechanism underlying the downregulation of TDP1 in ATL cells remains elusive. Here we identified the core promoter of the *TDP1* gene, which contains a conserved nuclear respiratory factor 1 (NRF-1) binding site. Overexpression of NRF-1 increased *TDP1*-promoter activity, whereas the introduction of dominant-negative NRF-1 repressed such activity. Overexpression of NRF-1 also upregulated endogenous TDP-1 expression, while introduction of shNRF-1 suppressed TDP1 in Jurkat T cells, making them susceptible to abacavir. These results indicate that NRF-1 is a positive transcriptional regulator of *TDP1*-gene expression. Importantly, we revealed that HTLV-1 bZIP factor (HBZ) protein which is expressed in all ATL cases physically interacts with NRF-1 and inhibits the DNA-binding ability of NRF-1. Taken together, HBZ suppresses TDP1 expression by inhibiting NRF-1 function in ATL cells. The HBZ/NRF-1/TDP1 axis provides new therapeutic targets against ATL and might explain genomic instability leading to the pathogenesis of ATL.

## Introduction

Adult T-cell leukemia (ATL) is a neoplastic disease of CD4+ T cells caused by human T-cell leukemia virus type 1 (HTLV-1)^1^. Because of its resistance to most chemotherapeutic agents, ATL has an extremely poor prognosis^2^. ATL occurs in ~5% of infected individuals after a long latency period in which Tax, one of the HTLV-1-encoded proteins, affects a wide variety of cellular-signaling pathways and plays a central role in leukemogenesis^3^. However, Tax expression is frequently undetectable in ATL cases because of genetic and epigenetic alterations such as mutations in the *tax* gene^4^, DNA methylation^5^, or deletion of the 5′ LTR^6^. By contrast, the *HTLV-1 bZIP factor (HBZ)* gene, which is encoded by the minus strand of the HTLV-1 provirus, is expressed in all ATL cases^7^. Suppression of *HBZ* gene transcription by short interfering RNA inhibits the proliferation of ATL cells^7^. HBZ also promotes the development of T-cell lymphomas and inflammatory diseases in transgenic mice^8^. This indicates that HBZ, in addition to Tax, plays an important role in the development of ATL.

We recently reported that abacavir (ABC), a nucleoside-analog reverse-transcriptase inhibitor, selectively kills ATL cells due to the downregulation of tyrosyl-DNA-phosphodiesterase 1 (TDP1), a DNA-repair enzyme^9^. TDP1 processes a wide range of substrates bearing 3′-blocking DNA (or RNA) lesions, including trapped topoisomerase I, chain-terminating nucleosides, and lesions caused by base alkylation^10–12^. Because of low TDP1 expression in ATL cells, once ABC is incorporated into genomic DNA it cannot be excised, leading to irreparable double-strand breaks in the cells. A recent study analyzing the 60 human-cancer cell lines of the NCI Developmental Therapeutic Anticancer Screen (the NCI-60) found two lung-cancer cell lines that do not express the TDP1 protein because one has a homozygous deleterious mutation and the other has a hypermethylated promoter of the *TDP1* gene^13^. However, the mechanism for impaired TDP1 expression in ATL cells has not been fully elucidated.

In this study, we show that nuclear respiratory factor 1 (NRF-1, also called α-pal) is a major transcriptional regulator of *TDP1*-gene expression and involved in the downregulation of TDP1, thereby enhancing the toxicity of ABC. NRF-1, a master regulator of mitochondria^14^, is reported as one of seven identified transcription factors whose binding sites are most frequently found in the proximal promoters of ubiquitously expressed genes^15^.

We also demonstrate that HBZ suppresses the transcription of *TDP1* by interfering with the DNA-binding activity of NRF-1. These results indicate that HBZ suppresses the NRF-1-mediated expression of *TDP1*, leading to the high susceptibility of ATL cells to ABC.

## Results

### Identification of NRF-1 as a regulator of *TDP1* transcription

**Figure 1:**
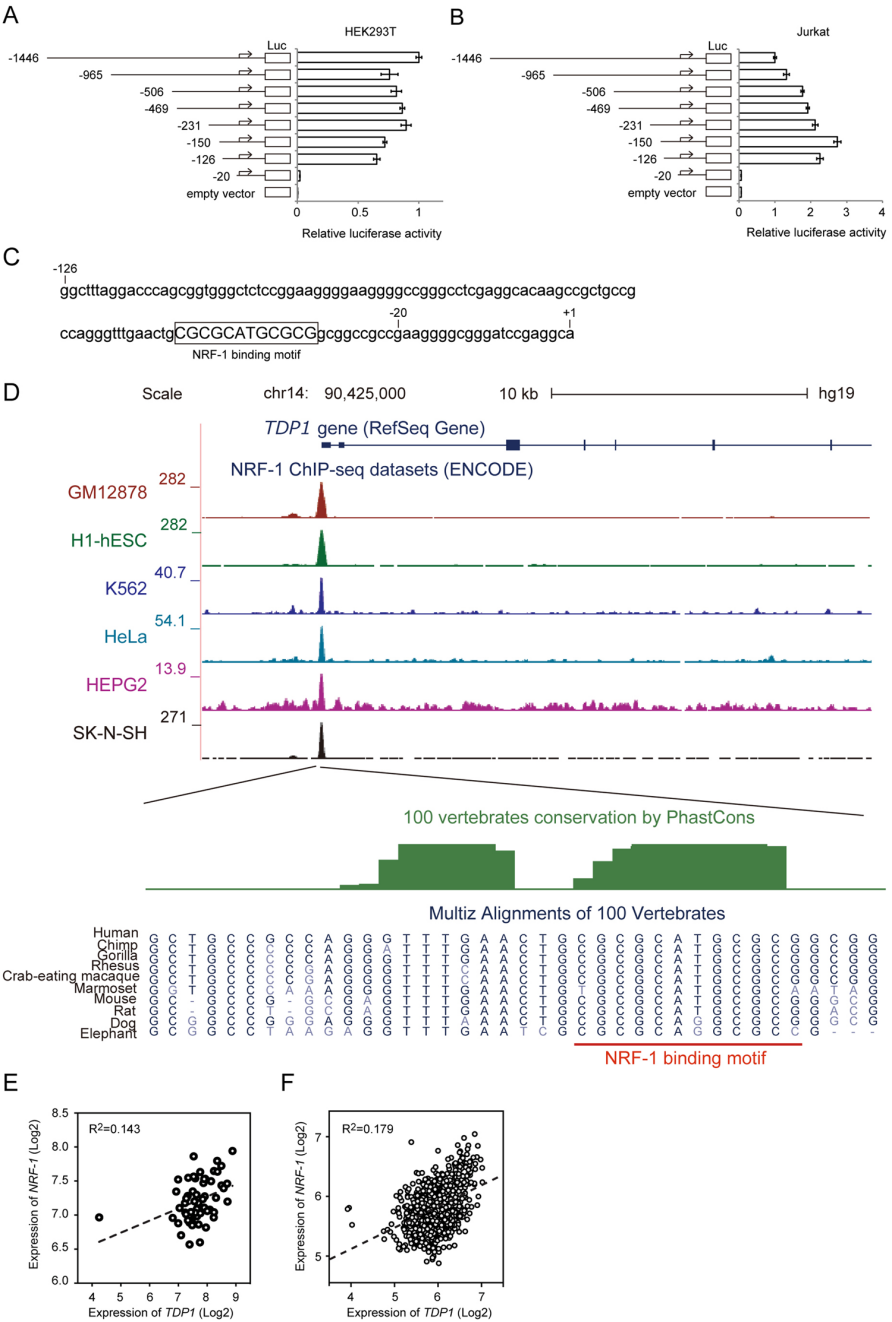
Identification of the core promoter of *TDP1*. (**A**) HEK293T cells and (**B**) Jurkat T cells were transfected with a luciferase-reporter vector driven by truncated *TDP1* promoter. Relative luciferase activity was calculated by comparison with the luciferase activity of the largest construct of TDP1-Luc (−1446/+193). Data shown are the mean ± SD (*n* = 3). (**C**) NRF-1-binding motif in the region between −126 and −20 of the *TDP1* promoter identified by a JASPAR database search (http://jaspar.genereg.net). The “CGCGCATGCGCG” in the square is an NRF-1-binding motif. (**D**) Illustration of the NRF1-binding site in the *TDP1* promoter. ENCODE ChIP-seq data for NRF-1 is shown on UCSC genome browser view (Kent *et al*. Genome Res. 2002 Jun;12(6):996-1006). Sequences surrounding the NRF-1 binding motif are zoomed in below. Phastcon vertebrate conservation (Siepel *et al*. Genome Res. 15, 1034–1050 (2005)) is shown in green, and NRF-1 binding motif is highlighted in red. (**E**) Correlation between the expression levels of *TDP1* and *NRF-1* was analyzed in the NCI-60 human-tumor cell-line panel. The broken line represents the regression curve (Y = 0.17X + 5.85). R^2^ = 0.143, *P* = 0.003, n = 59. (**F**) Correlation between the expression levels of *TDP1* and *NRF-1* was analyzed in the Cancer Cell Line Encyclopedia. The broken line represents the regression curve (Y = 0.37X + 3.60). R^2^ = 0.179, *P* < 0.0001, n = 1,036.

To search for the cause of the downregulation of TDP1 in ATL cells, we first investigated whether the *TDP1* gene was mutated or if its promoter was epigenetically modified in ATL cells. We detected no mutations in the *TDP1* gene and no promoter methylation in either the ED-40515(-) cell line or the MT-2 cell line (Supplementary Fig. S1). We next tested the promoter activity of *TDP1* in HEK293T cells and Jurkat T cells via a luciferase reporter assay, using various truncated *TDP1* promoter constructs. An analysis using the DataBase of Transcription Start Sites (DBTSS)^16^ revealed a *TDP1* transcription start site +45 nucleotides downstream of the site registered at NM_018319. We identified the region between −126 and −20 from the transcription start site of the *TDP1* promoter as the core promoter (Fig. 1A and B). A JASPAR database search (http://jaspar.genereg.net) showed that −126 to −20 of the *TDP1* promoter contains a predicted NRF-1-binding motif (Fig. 1C). Strikingly, a comparative genomics analysis of the promoter region containing the NRF-1-binding motif revealed high degree of sequence conservation, indicating the functionality (Fig. 1D). Furthermore, chromatin immunoprecipitation sequencing (ChIP-seq) dataset for NRF-1 from the ENCODE project^17^ showed NRF-1 binding to the *TDP1* promoter (Fig. 1D). We then investigated the correlation between the expression levels of *TDP1* and *NRF-1*. *TDP1* expression had a significant correlation with *NRF-1* expression in the NCI-60 human-tumor cell-line panel (Fig. 1E) as well as in the Cancer Cell Line Encyclopedia (Fig. 1F). In addition, *TDP1* gene expression positively correlated with *NRF-1* gene expression both in mouse and human as determined by the FANTOM5 gene expression atlas^18^, indicating the conserved mode of *TDP1* gene regulation by NRF-1 (Supplementary Fig. S2).

**Figure 2:**
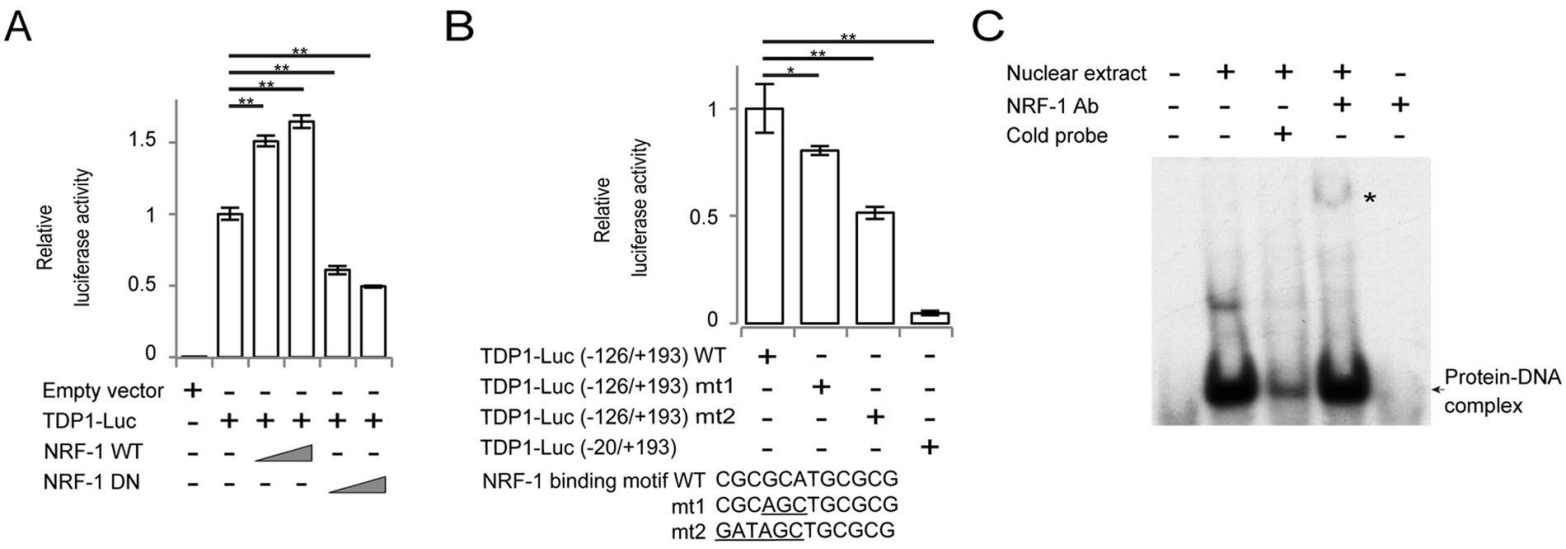
Identification of NRF-1 as a positive regulator of *TDP1*. (**A**) Effects of NRF-1 and its mutants on the luciferase activity of the *TDP1* promoter. 0.2 µg of TDP1-Luc (−126/+193) was transfected into HEK293T cells with or without vectors expressing wild-type (WT) (0.1 μg or 0.2 μg) or dominant-negative mutant (DN) (0.025 μg or 0.05 μg) of NRF-1. Relative luciferase activity was calculated by comparison with the basal luciferase activity of TDP1-Luc. Data shown are the mean ± SD (*n* = 3). (**B**) Effects of a mutated sequence of the NRF-1-binding motif on the luciferase activity of the *TDP1* promoter. Wild-type TDP1-Luc (−126/+193), mutated TDP1-Luc (−126/+193) (mt-1 and mt-2), or TDP1-Luc (−20/+193) was transfected into HEK293T cells. Relative luciferase activity was calculated by comparison with the basal luciferase activity of TDP1-Luc. Data shown are the mean ± SD (*n* = 3). (**C**) NRF-1 binds the promoter of *TDP1 in vitro*. A gel-shift assay was performed to analyze protein-DNA interactions. The NRF-1 protein-DNA complexes are indicated by the arrow. Supershift experiments were performed with anti-NRF-1 antibody.

**Figure 3:**
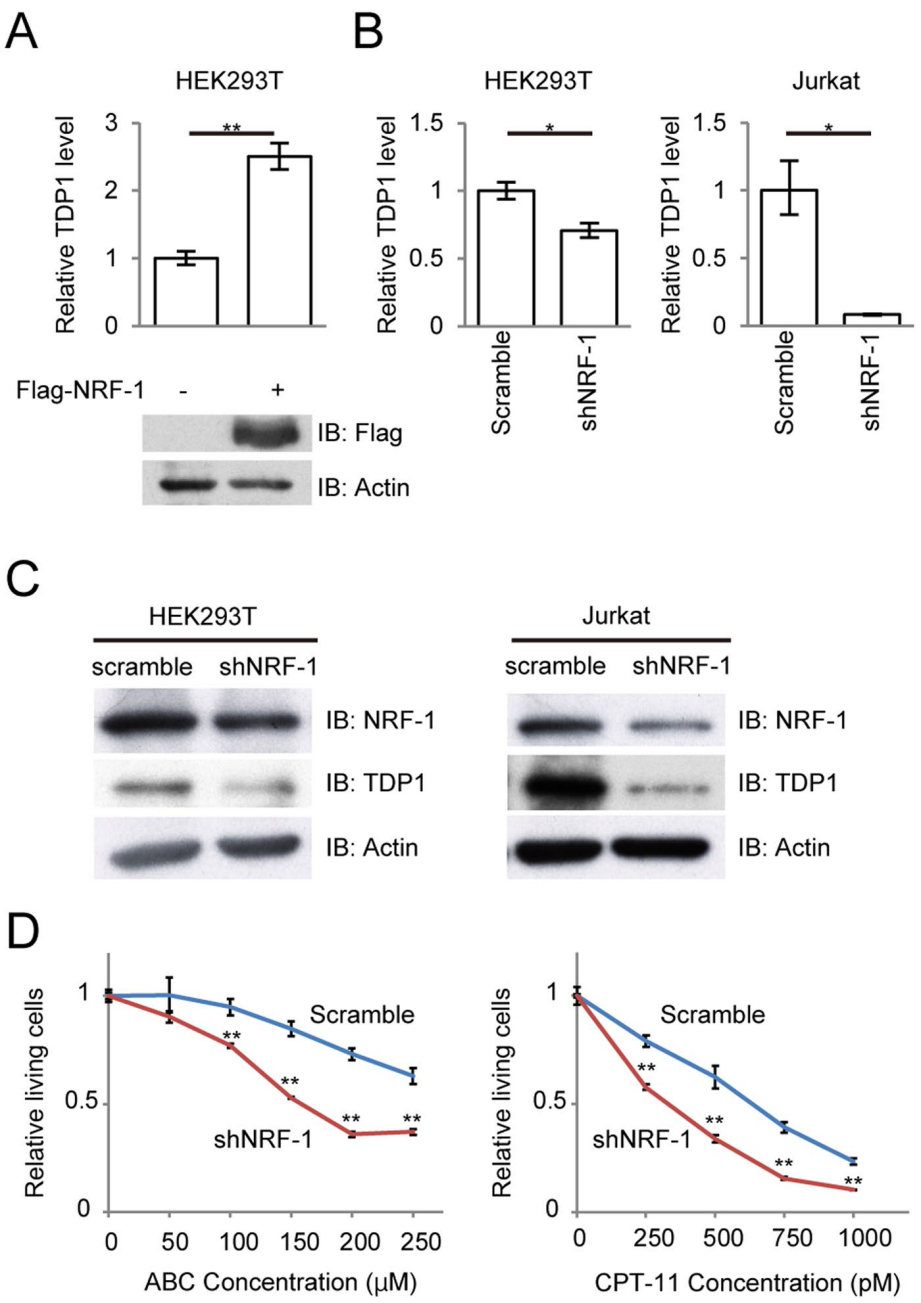
Functional analysis of the effect of NRF-1 on the expression level of TDP1. (**A**) Quantitative comparison of *TDP1* mRNA levels between NRF-1 ectopic expression and negative-control cells in HEK293T cells by real-time PCR. Transfectants were harvested 36 h after transfection of lentivirus vector expressing Flag-NRF-1. Data shown are the mean ± SD (*n* = 3). (**B**) and (**C**) Transfection of lentivirus vector expressing shNRF-1 decreases the expression of TDP1 mRNA and protein in HEK293T cells and Jurkat T cells. The transfectants were harvested 5 days after transfection. Data shown are the mean ± SD (*n* = 3). (**D**) Viability of the shNRF-1 transfected Jurkat T cells after 48 h treatment with the indicated concentration of ABC or CPT-11. MTS values of treated cells relative to untreated cells are shown. Data shown are the mean ± SD (*n* = 3).

To explore the effect of NRF-1 on *TDP1*-promoter activity, we transiently transfected NRF-1 wild-type (WT) or dominant negative (DN) mutant lacking the transactivation domain^19^ into HEK293T cells with a TDP1-Luc (−126/+193) reporter plasmid and performed a luciferase reporter assay. Overexpression of NRF-1 WT induced a significant dose-dependent increase in *TDP1*-promoter activity, while introduction of NRF-1 DN showed a dose-dependent decrease in the activity (Fig. 2A). In Jurkat T cells, introduction of NRF-1 DN showed a decrease in *TDP1*-promoter activity, while NRF-1 WT did not show significant increase in the activity (Supplementary Fig. S3), which can be explained by the abundant expression of NRF-1 in Jurkat T cells. Furthermore, mutations in the NRF-1-binding motif reduced *TDP1*-promoter activity (Fig. 2B). To demonstrate the ability of NRF-1 to bind to the *TDP1* promoter, we performed a gel-shift assay using nuclear extracts from HEK293T cells. The binding of NRF-1 to the probe was detected as a shift (Fig. 2C, arrow) and the specificity was confirmed by a super-shift experiment (Fig. 2C, asterisk). These results demonstrate that NRF-1 regulates the transcriptional activity of the *TDP1* promoter. To further assess the function of NRF-1 in endogenous *TDP1*-gene transcription, we performed overexpression as well as knockdown of NRF-1. Overexpression of NRF-1 upregulated the expression levels of the *TDP1* transcript in HEK293T cells (Fig. 3A), while knockdown of NRF-1 by shRNA downregulated the expression levels of both *TDP1* mRNA and protein in HEK293T cells and Jurkat T cells (Fig. 3B and C).

Since reduced TDP1 expression enhances susceptibility to ABC and CPT-11 as previously reported^9^, we measured the sensitivity of the NRF-1-knockdown Jurkat T cells to both drugs via MTS assay, demonstrating that the shNRF-1-transduced Jurkat T cells were more sensitive to ABC and CPT-11 than the control sh-transduced cells (Fig. 3D). In contrast, we found no significant differences in sensitivity to adriamycin, etoposide, or cisplatin—the repair pathways for which do not involve TDP1—between the NRF-1-knock-down Jurkat T cells and the control (Supplementary Fig. S4). These results indicate that NRF-1 is a major transcriptional regulator of *TDP1*-gene expression and affects sensitivity to ABC by downregulating TDP1 expression.

### HBZ suppresses *TDP1* expression by inhibiting NRF-1 activity

**Figure 4:**
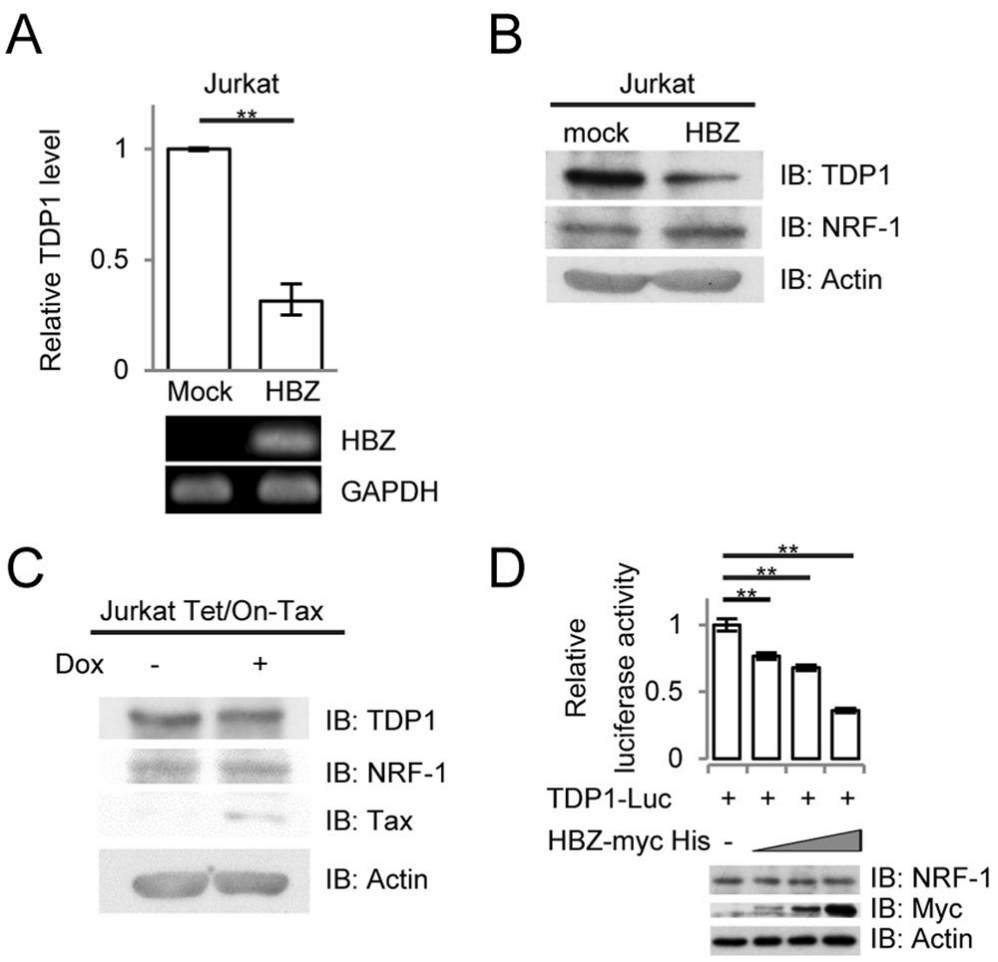
Functional analysis of the effect of the *HBZ* gene on the expression level of TDP1. (**A**) Comparison of *TDP1* mRNA transcription in the Jurkat T cells and the Jurkat-HBZ cells by real-time PCR. Data shown are the mean ± SD (*n* = 3). Expression of the *HBZ* gene in the Jurkat-HBZ cells was analyzed by RT-PCR. (**B**) Comparison of TDP1, NRF-1, and actin expression in the Jurkat T cells and the Jurkat-HBZ cells by immunoblotting. (**C**) Jurkat Tet/On-Tax cells were treated with doxycycline for 36 h. Expressions of TDP1, NRF-1, Tax, and actin are shown by immunoblotting. (**D**) Effects of HBZ on *TDP1*-promoter activity. 0.2 µg of TDP1-Luc (−126/+193) was transfected into HEK293T cells with or without vectors expressing HBZ (0.05, 0.1, or 0.2 µg). Relative luciferase activity was calculated by comparison with the basal-luciferase activity of TDP1-Luc. Data shown are mean ± SD (*n* = 3).

To better understand the mechanism of TDP1 downregulation in ATL cells, we examined the protein expression levels of NRF-1 in HTLV-1-infected cell lines ED-40515(-), MT-2, Hut-102, and ATL-43T, wherein *TDP1* expression was reduced compared to that of Jurkat T cells as we reported previously (Supplementary Fig. S5A). However, except for in Hut-102, NRF-1 expression was not downregulated in the cell lines tested (Supplementary Fig. S5B). We checked the sequence of *NRF-1* gene in these cell lines using Ensembl gene database and found no mutations. Since HTLV-1 viral proteins such as Tax and HBZ interact with many transcription factors and promote or attenuate their functions, we hypothesized that such proteins might also interact with NRF-1 and inhibit its transcriptional activity, resulting in the downregulation of TDP1. To investigate the effect of Tax and HBZ on TDP1 expression, we examined the expression of Tax and HBZ in cell lines tested. The expression of Tax protein was detected in MT-2 and Hut-102, and not in ED-40515(-) and ATL-43T (Supplementary Fig. S5B). The expression of *HBZ* was detected in all the HTLV-1-infected cell lines by real-time PCR (Supplementary Fig. S5C). Furthermore, we checked the expression levels of TDP1 in Jurkat Tax Tet-on cells and in HBZ-expressing Jurkat T (Jurkat-HBZ) cells. The expression of both TDP1 mRNA and protein was reduced in the Jurkat-HBZ cells, compared to the control Jurkat T cells (Fig. 4A and B, respectively), while Tax induction in the Jurkat T cells did not induce any significant changes in TDP1 expression (Fig. 4C). We therefore focused on a role of HBZ in the regulation of TDP1 expression by investigating the effect of HBZ on *TDP1*-promoter activity. As shown in Fig. 4D, HBZ clearly suppressed TDP1-promoter activity in a dose dependent manner.

**Figure 5:**
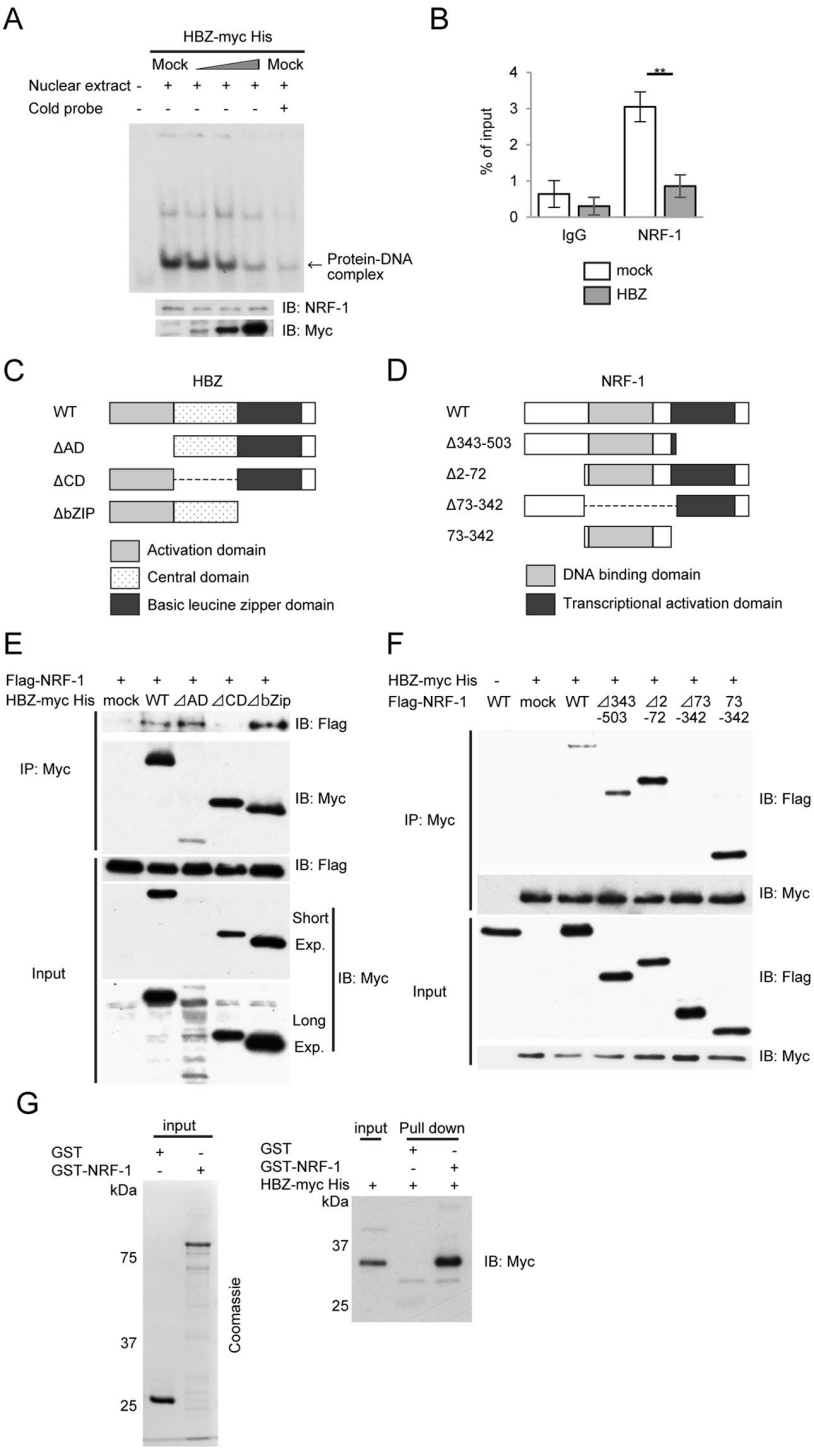
HBZ inhibits the DNA-binding activity of NRF-1 by physical interaction with NRF-1. (**A**) HBZ reduces NRF-1 binding to the NRF-1-binding motif in the TDP1 promoter. A gel-shift assay was used to analyze protein-DNA interactions. HEK293T cells were transfected with increasing amounts of HBZ (0, 1, 2, and 4 µg). Expression of NRF-1 and HBZ protein contained in the nuclear extracts was analyzed by immunoblotting. The position of the relevant protein-DNA complexes is indicated by the arrow. (**B**) Jurkat T cells and Jurkat-HBZ cells were immunoprecipitated with anti-NRF-1 antibody and quantified by real-time PCR. Data shown are the mean ± SD (n = 3). (**C**) Schema of *HBZ* gene. (**D**) Schema of *NRF-1* gene. (**E**) and (**F**) The expression vectors of the indicated proteins were co-transfected into HEK293T cells, and their interactions were analyzed by co-immunoprecipitation assay. (**G**) NRF-1 was purified from *E. coli* as a GST fusion protein. GST alone or GST-NRF-1 was incubated with *in vitro* transcribed/translated HBZ-mycHis. GST pull-downs and input were subjected to Western blotting with anti-Myc antibody.

We next investigated the effect of HBZ on the DNA-binding activity of NRF-1. A gel-shift assay showed a dose-dependent inhibition by HBZ proteins on the formation of the DNA/NRF-1 complex (Fig. 5A). We then performed ChIP-qPCR assays to determine whether HBZ interfered with the binding of NRF-1 to the *TDP1* promoter. The amount of NRF-1 binding sites precipitated with NRF-1 protein was decreased in the Jurkat-HBZ cells compared to that in control Jurkat T cells (Fig. 5B). These data show that HBZ suppresses NRF-1-mediated *TDP1* expression by inhibiting the DNA binding of NRF-1.

**Figure 6:**
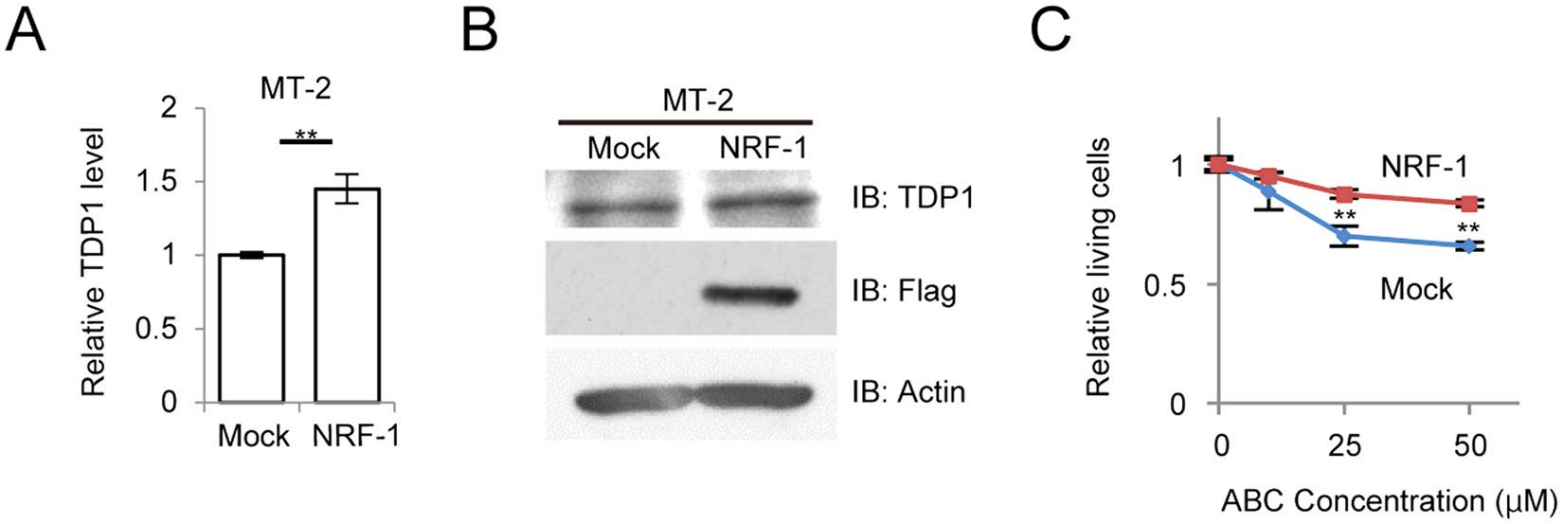
Overexpression of NRF-1 overcomes the inhibitory effect of HBZ on TDP1 expression. (**A**) Quantitative comparison of *TDP1* mRNA levels between Flag-NRF-1 ectopic expression and mock in MT-2 cells by real-time PCR. Data shown are the mean ± SD (*n* = 3). (**B**) Comparison of TDP1-protein expression between Flag-NRF-1 ectopic expression and mock in MT-2 cells by immunoblotting. (**C**) Viability of the Flag-NRF-1-overexpressed MT-2 cells and control cells after 36 h treatment with the indicated concentration of ABC. MTS values of treated cells relative to untreated cells are shown. Data shown are the mean ± SD (*n* = 3).

We next examined the interaction between HBZ and NRF-1 via co-immunoprecipitation assays. HBZ consists of three domains: an activation domain (AD), a central domain (CD), and a basic leucine-zipper domain (bZIP). NRF-1 possesses two known domains: a DNA-binding domain and a transcriptional-activation domain. Accordingly, we tested three HBZ deletion mutants (ΔAD, ΔCD, and ΔbZIP) (Fig. 5C) and four NRF-1 deletion mutants (Δ343-503 [the dominant negative mutant as shown in Fig. 2C], Δ2–72, Δ73–342, and 73–342) (Fig. 5D). In the HBZ-deletion mutants, the central domain of HBZ is essential for binding to NRF-1 (Fig. 5E), while in the NRF-1-deletion mutants, the DNA-binding domain of NRF-1 is indispensable for binding to HBZ (Fig. 5F). We further performed gel-shift assays using the nuclear extracts from HEK293T cells transiently transfected with HBZ-WT as well as with HBZ-ΔCD (which does not bind to NRF-1). The HBZ-WT protein inhibited the formation of the DNA/NRF-1 complex, whereas HBZ-ΔCD did not (Supplementary Fig. S6). To confirm direct interaction between NRF-1 and HBZ, *in vitro* GST pull-down assay was performed. GST-tagged NRF-1 was able to interact with HBZ-mycHis but GST was not (Fig. 5G). Since HBZ functions as both RNA and protein^7^, we examined whether HBZ RNA or protein inhibited the transcriptional activity of NRF-1 by conducting a luciferase assay using the *HBZ* mutant, in which all coding regions were replaced with silent mutations (HBZ-SM). HBZ-SM suppressed the activation of TDP1-Luc, as did HBZ-WT (Supplementary Fig. S7). These findings indicate that the HBZ protein, not RNA, suppressed the transcription of *TDP1* by interacting with the DNA-binding domain of NRF-1 and interfering with the DNA-binding activity of NRF-1. Furthermore, we sought to determine if the overexpression of NRF-1 could overcome the inhibitory effect of HBZ on TDP1 expression. Overexpression of Flag-NRF-1 WT in MT-2 cells upregulated the expression level of both the *TDP1* transcript (Fig. 6A) and the TDP1 protein (Fig. 6B). More importantly, NRF-1-overexpressed-MT-2 cells were more resistant to ABC than were the control cells (Fig. 6C). We thus conclude that HBZ downregulates the expression of TDP1 in HTLV-1-infected cells by interacting with NRF-1 and inhibiting its transcriptional activity.

Finally, we performed differential gene expression analysis using microarray datasets for acute-type ATL cells derived from patients and normal CD4^+^ T lymphocytes^20^ and showed that NRF-1 binding motif occurred more frequently in the promoter regions of the downregulated genes in ATL cells (Odds ratio: 1.17, *P*-value: 0.045). This finding further implicates a more general role of NRF-1 in ATL development by affecting the transcription of multiple genes.

## Discussion

We show in this study that HBZ inhibits *TDP1*-gene expression by targeting NRF-1. First, we demonstrate for the first time that a transcription factor NRF-1 regulates *TDP1* transcription. Since the NRF-1-binding motif is often found in a large number of TATA-less promoters around the transcriptional-start site, it has been suggested that NRF-1 plays a major role as a proximal promoter-binding factor. We find that the promoter of *TDP1*, which is TATA-less, contains a conserved NRF-1-binding motif in the region between −126 and −20, and that NRF-1 functions as a key transcription factor for *TDP1* gene. TDP1 processes 3′- and 5′-DNA ends by excising irreversible protein-tyrosyl-DNA complexes involving topoisomerase I^21^. DNA topoisomerases are crucial to the regulation of the topology of the genome and for removing DNA supercoiling resulting from transcription, replication, and chromatin dynamics. TDP1 has also been implicated as a backup repair pathway for the repair of topoisomerase-II-cleavage complexes^22^ and as a regulator for the fidelity of non-homologous end-joining^23^. Thus, TDP1 contributes to genomic stability. Indeed, the loss of Tdp1 function affects mutation rates at repeat sequences in yeast^24^. These suggest that the reduced expression of TDP1 might contribute to genomic instability often seen in ATL cells.

NRF-1 was previously identified as a transcriptional regulator of genes involved in mitochondrial biogenesis^25^. NRF-1 also plays a major role in regulating and coordinating various genes involved in embryonic development^26^, cell proliferation, and DNA synthesis and repair^14,27^. However, the pathogenic role of NRF-1 in cancer cells remains unclear. NRF-1 expression is selectively upregulated in human breast cancer cells relative to adjacent stromal tissue, which correlates with metastasis and poor prognosis^28^. Upregulation of NRF-1 is also observed in type-I-endometrial cancer^29^ and thyroid oncocytoma^30^. Conversely, downregulation of NRF-1 mediated by miR-504 affects the radioresistance of nasopharyngeal carcinoma^31^. In the present study, we show that the function of NRF-1 is attenuated by HBZ in HTLV-1-infected cells, which results in reduced expression of TDP1. Furthermore, we show that NRF-1 motif was enriched in the promoter regions of the downregulated genes in ATL cells, implicating that HBZ might contribute to ATL development by affecting transcription of multiple NRF-1 target genes. Moreover, HBZ interacts with many transcription factors, including CREB^32^, c-Jun^33^, JunB^33^, JunD^34^, MafB^35^, ATF3^36^, and FoxO3a^37^ acting as a hub for complex transcriptional network. This indicates a pathological role of the HBZ viral protein in global gene dysregulation in ATL cells by affecting the expression of numerous downstream target genes. In this study, HTLV-1-infected cell lines we used showed various expression levels of *HBZ* mRNA, which is almost the same result with previous report^38^. In the same report, HBZ protein expression was fully detectable in all the cell lines, even in MT-2 and Hut-102 that showed low *HBZ* mRNA expression.

In addition to the various roles that HBZ plays in the pathogenesis of ATL, repression of NRF-1 may also be important to ATL development. That is because, besides regulating TDP1 expression, NRF-1 targets other genes involved in various DNA-repair pathways such as non-homologous end-joining, base-excision repair, and mismatch repair^39^. A recent study involving the whole-genome sequencing of primary ATL found more extent structural variations of chromosomes than for other hematological malignancies^40,41^, a finding that indicates genomic instability^42^. The mechanisms that induce genomic instability in ATL have not yet been fully elucidated, but it has been shown that HBZ promotes onco-miR expression as well as DNA-strand breaks by downregulating the expression of the OBFC2A protein via posttranscriptional activation of miR17 and miR21, resulting in both genetic instability and cell proliferation^43^. We postulate that the impaired NRF-1 activity by HBZ may be another mechanism by which genomic instability is induced and which may also contribute to the development of ATL.

In conclusion, we show herein that NRF-1 is a major transcriptional regulator of *TDP1*-gene expression and that HBZ suppresses the NRF-1-mediated transcription of TDP1. These findings provide novel insights into the pathogenesis of ATL and point to the development of a novel therapeutic strategy against the disease.

## Materials and Methods

### Cell culture and human-cell-killing assay

HTLV-1-infected T-cell lines (ED-40515(−), MT-2, Hut-102, and ATL-43T) and non–HTLV-1–infected T-cell line (Jurkat T cell) were cultured in RPMI1640 (Nacalai Tesque) containing 10% FBS and 1% PSG (Invitrogen). Jurkat T cells stably expressing a spliced form of HBZ called Jurkat-HBZ were maintained as described previously^44^. HEK293T cells were maintained with DMEM (Nacalai Tesque) containing 10% FBS and 1% PSG. The Jurkat Tet/On-Tax cells^45^ kindly gifted from Dr. W.C. Greene were maintained in RPMI1640 supplemented with 10% tetracycline-free FBS (Clontech) and treated with 1 µg/ml doxycycline for 36 hours for inducible Tax expression. Human-cell-killing assay was performed as described previously^9^.

### Reagents and antibodies

ABC was purchased from Carbosynth (NA10019). CPT-11 was purchased from TopoGEN (TG4110), cisplatin was purchased from Nihon-kayaku (Randa), etoposide was purchased from TREVIGEN (4886–400–01), and adriamycin was purchased from Kyowa Hakko Kirin (Adriacin). These chemicals were dissolved in 100% dimethyl sulfoxide (Nacalai Tesque). Antibodies used were as follows: anti-FLAG M2 (F9291, Sigma-Aldrich), anti-Myc (C3956, Sigma-Aldrich), anti-NRF-1(H-300, sc-33771, Santa Cruz), anti-TDP1 (ab4166, Abcam), and anti–β-actin (AC-15, A5441, Sigma-Aldrich). Anti-Tax (Lt-4) antibody was as described previously^46^.

### Quantitative real-time PCR and western blot

Quantitative real-time PCR was performed as described previously^9^. The primers used were TDP1 forward, 5′-AGGCAGCCTTGGACAGATT-3′; TDP1 reverse, 5′-GGTCAGCTGAGACTTCTGGC-3′; HBZ forward, 5′-ATGGCGGCCTCAGGGCTGTT-3′; HBZ reverse, 5′-GCGGCTTTCCTCTTCTAAGG-3′, GAPDH forward, 5′-GAAGGTGAAGGTCGGAGTC-3′; and GAPDH reverse, 5′-GAAGATGGTGATGGGATTTC-3′. Western blot was performed as described previously^47^.

### Plasmid constructs

The sequence encoding −1446/+193 bp of the *TDP1* promoter was generated by PCR amplification using genomic DNA from a healthy donor. The PCR fragments was subcloned into pGL3-basic (Promega), and the vectors encoding truncated *TDP1* promoter were generated by PCR. The vectors encoding FLAG-NRF-1 WT, FLAG-NRF-1 DN and GST-NRF-1 were kind gifts from Dr. H. Izumi^19^. Δ2–72, Δ73–342, and 73–342 truncated form were generated by PCR. The vectors encoding the myc-His-tagged form of HBZ-WT and its mutants (HBZ-ΔAD and HBZ-ΔCD, HBZ-ΔbZIP) have been described previously^44^. The vector encoding the myc-His-tagged form of HBZ-SM was generated by subcloning the coding sequence of HBZ-SM^7^ into pcDNA3.1 (−)/myc-His.

### Luciferase reporter assay

HEK293T cells were grown in 24-well plates (1 × 10^5^ cells/well). The following day, cells were co-transfected with 0.5 µg of luciferase-reporter plasmid and other protein-expressing vectors and 0.5 ng of the control *Renilla* luciferase reporter (phRL-TK) using the X-tremeGENE HP DNA Transfection Reagent (Roche). For Jurkat T cells, 10 μg of the reporter plasmid and 0.1 μg of *Renilla* luciferase reporter were transfected by electroporation into 1 × 10^7^ cells. Twenty-four hours later, cells were harvested and luciferase activity was measured with a Dual-Luciferase Reporter Assay System (Promega). Relative luciferase activity was calculated as the ratio of firefly to *Renilla* luciferase activity.

### Lentiviral vector construction and transfection of the recombinant lentivirus

FLAG-NRF-1 was cloned into the lentiviral vector CSII-CMV-MCS-IRES-hrGFP. HEK293T cells and MT-2 cells were infected with recombinant lentivirus as described^47^, and confirmation of infectivity was based on GFP expression. We modified psicoR-mCherry vector for delivery of anti-NRF-1 short hairpin RNAs (shRNA) to Jurkat T cells. The shRNA sequence used was 5′-CATATGGCTACCATAGAAG-3′,which has been previously reported^48^. The NRF-1-expression level in the infected Jurkat T cells was verified by western blot.

### Co-immunoprecipitation assay

Six-well plate HEK293T cells were transfected with expression plasmids using the X-tremeGENE HP DNA Transfection Reagent (Roche). At 48 h post transfection, cells were lysed in 800 µl of lysis buffer (25 mM Tris–HCl [pH 8.0], 100 mM NaCl, 0.1% NP-40, 1 mM EDTA, and phosphatase-inhibitor cocktail). Lysates were incubated with the desired antibody for 1 h at 4 °C. Finally, the antibody complexes were captured with protein A-sepharose beads for 1 h. Beads were washed four times with lysis buffer and immunoprecipitants were eluted and analyzed by western blot.

### Chromatin-immunoprecipitation (ChIP) analysis

A ChIP assay was performed using an EpiQuik TM Chromatin Immunoprecipitation Kit (Epigentek) following the protocol provided by the supplier. 2 × 10^6^ Jurkat-HBZ cells and control cells were used in each assay. The sonicated samples were immunoprecipitated with the NRF-1 antibody (ab34682, Abcam) or normal rabbit IgG (sc-2027 X, Santa Cruz) overnight at 4 °C. Protein-DNA complexes were de-crosslinked at 65 °C for 6 h. Primers used for the quantification of the target fragments by real-time PCR were 5′-TGCCGCCAGGGTTTGAA-3′ and 5′-CTGAGGCGCACAGAACCAAC-3′, which were constructed to contain the NRF-1-binding sequence located in −41/−30 bp of the *TDP1*-gene promoter. Individual PCRs were carried out in triplicate, and mean *C*t values were collected.

### Bisulfite genomic sequencing

Sodium-bisulfite treatment of genomic DNA was performed as described previously^13^. The primer sequences used to amplify the region at chr14 (90,422,115–90,422,835) (hg19) containing the CpG island (chr14:90,422,143-90,422,593) at the *TDP1* promoter were 5′-AGTTAGGAGAGATTAGGTTTTTTTAGTTT-3′ and 5′-ACAACAACTACTAACCTTACTACGTA-3′. PCR products were purified, cloned into pGEM-T Easy vector (Promega), and sequenced using the ABI PRISM 3130 Genetic Analyzer. For CpG-methylation analysis, a web-based bisulfite-sequencing-analysis tool called QUMA (quantification tool for methylation analysis) was used^49^.

### Electrophoretic mobility gel shift assays (EMSAs)

The second generation DIG Gel Shift kit (Roche) was used according to the manufacturer’s instructions. 10-cm dish HEK293T cells are transfected with 6 µg of HBZ-WT and HBZ-ΔCD expressing vectors. The sequences used for each EMSA oligonucleotide were 5′-ACTGCGCGCATGCGCGGCGG-3′ and 3′-CCGCCGCGCATGCGCGCAGT-5′. For the competition studies, excess (250-fold) unlabeled oligos were added to the reaction. NRF-1 antibody (ab34682, Abcam) was used for the supershift assay.

### *In vitro* binding assay

HBZ-mycHis protein was synthesized *in vitro* using the TNT T7 Quick Coupled Transcription/Translation System (Promega). GST and GST-NRF-1 proteins were produced in *E. coli* BL21 and purified with glutathione Sepharose 4B beads (GE Healthcare). The beads were incubated with HBZ-mycHis at 4 °C for 2 hr. The beads were washed and proteins were eluted, followed by Western blotting with anti-Myc antibody (C3956, Sigma-Aldrich).

### Microarray data processing

Microarray raw data for normal CD4^+^ T lymphocytes and acute-type ATL cells were retrieved from GEO accession GSE43017^20^. Robust multi-array average background correction and quantile normalization was applied using affyR Bioconductor packages^50^. Differential gene expression analysis was conducted using the limma Bioconductor R package^51^.

### Motif enrichment analysis

DNA Sequences (−2000 to +400 nt) for RefSeq genes were retrieved from https://genome.ucsc.edu/cgi-bin/hgTables. We counted the frequency of ENCODE NRF-1-binding motif (“GCGCNNGCGC”)^52^ occurring in this promoter regions for the downregulated genes in ATL cells (n = 997) and in control genes (n = 13,218). Enrichment of the motif was calculated by Fisher’s exact test. *P*-value was calculated using the Fisher’s Exact Test for the enrichment of the NRF-1 motif in the promoter regions of downregulated genes in ATL cells.

### Correlation analysis

The FANTOM5 Phase 1 promoter-level expression data for human and mouse were retrieved on 27/3/2017 from http://fantom.gsc.riken.jp/data/ ^18^. Promoter-level expression data was converted into gene-level expression data by summing each promoter-level expression mapping to the same gene.

### Statistical analyses

Statistical analyses were performed using the Student’s *t* test. Asterisks indicate significance (*p < 0.05, **p < 0.01).

### Data availability

The datasets analysed during the current study are available from the corresponding author on reasonable request.

## Electronic supplementary material

Supplementary Figures
