# Supplementary material for: HTLV-1 bZIP factor suppresses TDP1 expression through inhibition of NRF-1 in adult T-cell leukemia
Source: Sci Rep. 2017 Oct 9;7:12849. doi: 10.1038/s41598-017-12924-0 (PMC5634466; doi:10.1038/s41598-017-12924-0)
Supplement: Supplementary file 1 — Supplementary Figures [file 41598_2017_12924_MOESM1_ESM.pdf]

HTLV-1 bZIP factor suppresses TDP1 expression through inhibition of NRF-1 in adult T-cell leukemia

Yoko Takiuchi[1], Masayuki Kobayashi[1, *], Kohei Tada[1], Fumie Iwai[1], Maki Sakurada[1], Shigeki Hirabayashi[1], Kayoko Nagata[1], Kotaro Shirakawa[1], Keisuke Shindo[1], Jun-ichirou Yasunaga[2], Yasuhiro Murakawa[3], Vinodh Rajapakse[4], Yves Pommier[4], Masao Matsuoka[5] and Akifumi Takaori-Kondo[1]

[1]Department of Hematology and Oncology, Graduate School of Medicine, Kyoto University, 54 Shogoin-kawaracho, Sakyo-ku, Kyoto 606-8507, JAPAN

[2]Laboratory of Virus Control, Institute for Frontier Life and Medical Sciences, Kyoto University, 53 Shogoin-kawaracho, Sakyo-ku, Kyoto 606-8507, JAPAN

[3]RIKEN Preventive Medicine and Diagnosis Innovation Program, 1-7-22 Suehiro-cho, Tsurumi-ku, Yokohama, Kanagawa, 230-0045, JAPAN

[4]Developmental Therapeutics Branch and Laboratory of Molecular Pharmacology, Center for Cancer Research, National Cancer Institute, National Institutes of Health, Bethesda, MD 20892, USA

[5]Department of Hematology, Rheumatology and Infectious Disease, Kumamoto University Graduate School of Medicine, 1-1-1 Honjo, Kumamoto 860-8556, JAPAN

Supplementary Figure and Figure Legend

Supplementary Figure S1

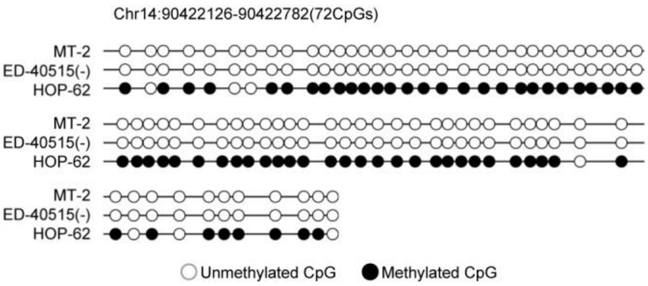

Supplementary Figure S1. DNA methylation in the promoter region of the *TDP1* gene in HTLV-1-infected cell lines ED-40515(-) and MT-2 and in the positive-control cell line (HOP-62). The chromosomal region (Chr14: 90422126-90422782) was sequenced. Non-methylated CpG dinucleotides are represented by white circles, and methyl-CpG dinucleotides by black circles.

Supplementary Figure S2

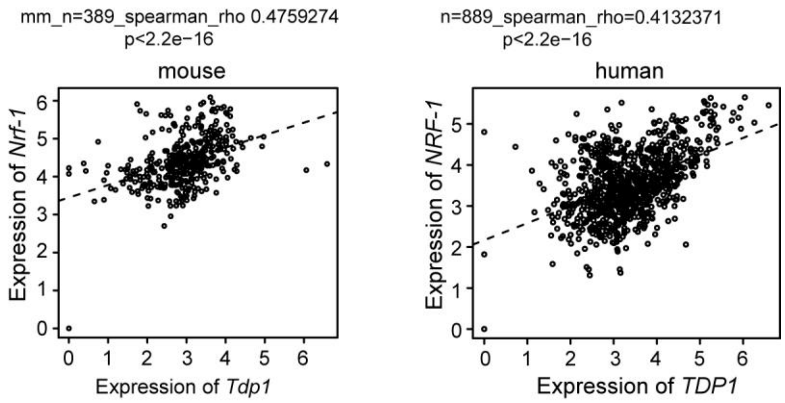

Supplementary Figure S2. Correlation between the gene expression levels of *TDP1*(*Tdp1*) and *NRF-1*(*Nrf-1*) across 889 human (right) and 389 mouse (left) samples was analyzed in the FANTOM5 Phase 1 data (mouse: Spearman rho 0.476, $P < 0.0001$; human: Spearman rho 0.413, $P < 0.0001$).

Supplementary Figure S3

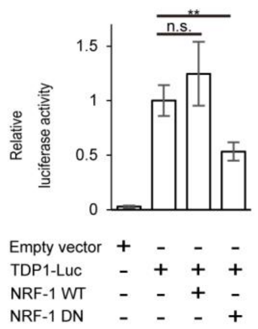

Supplementary Figure S3. Effects of NRF-1 and its mutants on the luciferase activity of the *TDP1* promoter. 5 µg of TDP1-Luc (-126/+193) was transfected by electroporation into 1 × 10^7 Jurkat T cells with or without vectors expressing wild-type (WT) (1.5 □g) or dominant-negative mutant (DN) (0.5 □g) of NRF-1. Relative luciferase activity was calculated by comparison with the basal luciferase activity of TDP1-Luc. Data shown are the mean ± SD (*n* = 3).

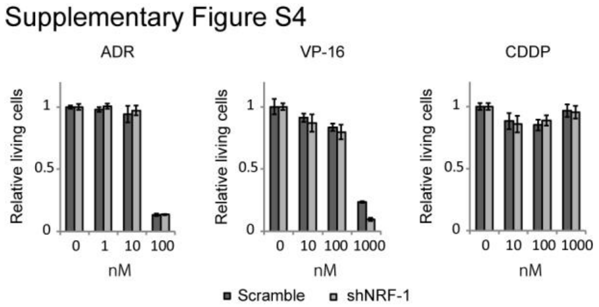

Supplementary Figure S4. Viability of shNRF-1 transfected Jurkat T cells after 48 h treatment with the indicated dose of adriamycin (ADR), etoposide (VP-16) and cisplatin (CDDP). MTS values of treated cells relative to untreated cells are shown. Data shown are the mean ± SD (n = 3).

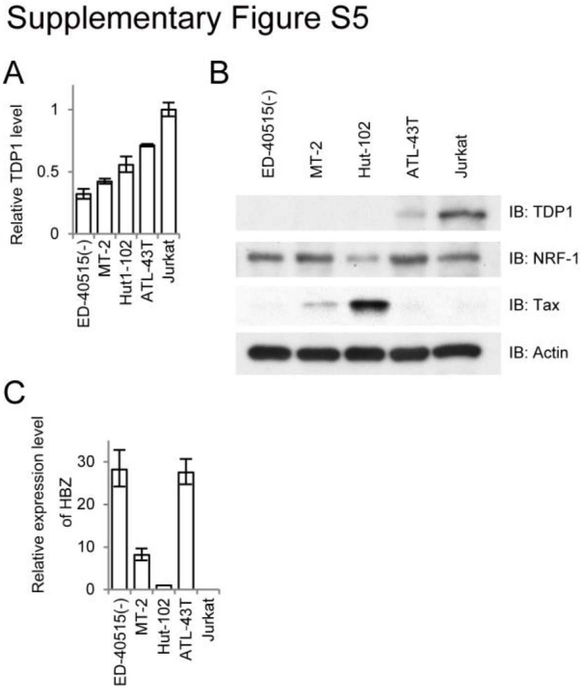

Supplementary Figure S5. Comparison of *TDP1* gene expression in HTLV-1-infected cell lines. (A) Comparison of *TDP1* mRNA expression in HTLV-1-infected cell lines by real-time PCR. Data shown are the mean ± SD (*n* = 3). (B) Comparison of TDP1, NRF-1, and Tax protein expression in HTLV-1-infected cell lines by immunoblotting. (C) Comparison of *HBZ* mRNA expression in HTLV-1-infected cell lines by real-time PCR. Data shown are the mean ± SD (*n* = 3).

Supplementary Figure S6

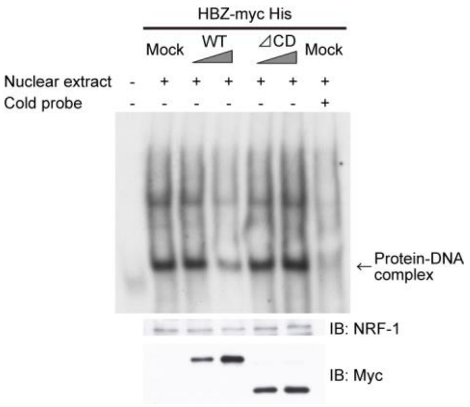

Supplementary Figure S6. HBZ reduces NRF-1 binding to the NRF-1 binding motif in the TDP1 promoter through the central domain (CD). A gel-shift assay was performed to analyze protein-DNA interactions. HEK293T cells were transfected with increasing amounts of HBZ wild-type (WT) and HBZ-ΔCD. Expression of the NRF-1 and HBZ (WT and ΔCD) proteins contained in the nuclear extracts were analyzed by immunoblotting. The positions of the protein-DNA complexes are indicated by the arrow.

Supplementary Figure S7

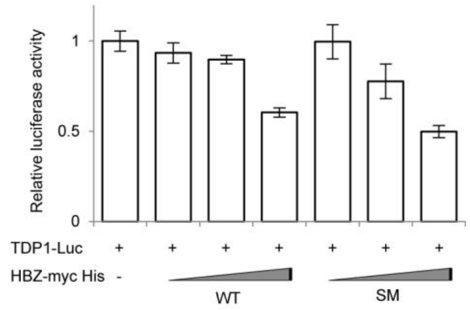

Supplementary Figure S7. Effects of *HBZ* and its mutants on *TDP1* promoter activity. Luciferase reporter (TDP1-Luc) was transfected into HEK293T cells with or without vectors expressing wild-type (WT) or mutated *HBZ* (SM) (0.05, 0.1, or 0.2 μg). Relative luciferase activities were calculated in comparison with the basal luciferase activity of TDP1-Luc. Data shown are the mean ± SD (n = 3).
